# The effect of peer victimisation on cognitive development in childhood: evidence for mediation via inflammation

**DOI:** 10.1007/s00127-025-02836-0

**Published:** 2025-03-17

**Authors:** Ellie Roberts, Marta Francesconi, Eirini Flouri

**Affiliations:** 1 Department of Arts and Sciences, University College London, Malet Place, London, NW1 6AP UK; 2 Department of Psychology and Human Development, Institute of Education, University College London, 20 Bedford Way, London, WC1H 0AL UK

**Keywords:** Peer victimisation, Cognition, Inflammation, Stress, ALSPAC

## Abstract

**Purpose:**

Peer victimisation, often a serious childhood stressor, has been associated with poor cognitive outcomes. The current study sought to uncover whether peer victimisation is associated with poor cognitive functioning in childhood via inflammation.

**Methods:**

Data from 4583 participants in the Avon Longitudinal Study of Parents and Children (ALSPAC) were analysed. Path analysis was conducted to determine whether inflammation, measured using IL-6 and CRP levels (age 9), mediates the effects of peer victimisation (age 8), even after controlling for other stressors, on multiple cognitive outcomes, including working memory (age 10), reading (accuracy, speed, and comprehension) (age 9), spelling (age 9), response inhibition (age 10), attentional control (age 11), and selective attention (age 11).

**Results:**

IL-6 and CRP partially mediated the effects of peer victimisation on working memory, reading accuracy, and selective attention. IL-6 partially mediated the effect of peer victimisation on reading comprehension, while CRP partially mediated the effect of peer victimisation on reading speed. All effects were small. Inflammation did not mediate the effects of peer victimisation on spelling, response inhibition or attentional control.

**Conclusion:**

Peer victimisation may impact on some aspects of children’s cognitive functioning via inflammation. The cognitive outcome specificity observed warrants further research.

**Supplementary Information:**

The online version contains supplementary material available at 10.1007/s00127-025-02836-0.

## Introduction

Peer victimisation is a form of bullying which describes the experience of being repeatedly maltreated by peers, and is often highly stressful and chronic [4, 44]. It is also not uncommon. For example, in both the United States and United Kingdom, peer victimisation is the most prevalent form of abuse in those under the age of 25 [40]. Importantly, it is strongly associated with poor physical and mental wellbeing, both concurrently and prospectively [2, 50], but also poor cognitive functioning. For example, a monozygotic twin study by Carroll et al. [11], controlling for genetic and familial factors, found that peer victimisation was associated with differences in selective attention in childhood. More recently, Menken et al. [34] demonstrated in a large cross-sectional study that parent-reported peer victimisation was associated with impaired memory, language, cognitive control, attention, and information processing, in children. Together with findings that stressors are in general related to impairments in learning, memory and other executive functions in adults [33, 42], these studies suggest that peer victimisation may be a particularly salient risk factor for poor cognitive functioning in childhood.

Few studies however have investigated biological processes as possible mediators of such a link in childhood. Inflammation has previously been suggested to impact cognition through effects on neurotransmission, synaptic plasticity and branching of dendrites [53], with one study demonstrating an association between inflammation and smaller hippocampal volume in the elderly [45]. The effect of inflammation on cognition in young populations has received less attention, although Cullen et al. [14] found that higher levels of inflammation correlated with poorer cognitive function in adolescents aged 11–14, and Jonker et al. [25] demonstrated a prospective association between inflammatory markers at age 16 and cognitive function at age 18. Therefore, it is possible that inflammation, associated with childhood stressors in general [7, 18], but also peer victimisation specifically [13, 46], could be a mediator. Inflammation has also been found to mediate the effect of stressful life events on child mental health [19], in turn highly correlated with cognitive function in childhood [24].

### The current study

The current study tested the hypothesis that inflammation may mediate the effect of peer victimisation on child cognitive functioning. It used a prospective general-population sample of children, a considerable strength over the existing research on inflammation and cognition, which has typically focussed on elderly or clinical populations [35, 47]. Furthermore, unlike most studies on the link between cognition and inflammation which have predominantly focused on memory [1], it measured cognitive functioning across several domains. Finally, it controlled for multiple potential confounders, notably other stressors experienced across childhood, thus enabling the ascertainment of both stressor and outcome specificity. Its focus was experiences of overt peer victimisation (direct interactions i.e., physical or verbal abuse), rather than relational peer victimisation (indirect interactions such as social exclusion or rumour spreading), as overt peer victimisation is more common before adolescence and is generally associated with poorer outcomes [12].

## Method

### Participants

The study used data from the Avon Longitudinal Study of Parents and Children (ALSPAC), a prospective population-based study investigating social, psychological, and biological factors which may affect child development and wellbeing [8], http://www.bristol.ac.uk/alspac/researchers/our-data for details). From April 1991 to December 1992, ALSPAC recruited 14,541 women in Bristol, UK. Parents completed questionnaires about their own and their child’s wellbeing from the first pregnancy trimester onwards. From age 7, children were invited to attend annual clinical assessments, during which they underwent physical and psychological tests [20]. At the age of 12 months, 13,988 children were still alive, and the development of these children has since been followed [22]. Of the original 14,541 initial pregnancies 338 were from women who had already enrolled with a previous pregnancy, therefore 14,203 unique mothers were initially enrolled in the study. Following additional recruitment, 630 more women who did not enrol originally have provided data since their child was age 7, increasing the number to 15,447 pregnancies and 14,901 children alive at 12 months of age [20]. Consent for biological samples has been collected in accordance with the Human Tissue Act 2004, and ethical approval for the study was obtained from the ALSPAC Ethics and Law Committee and Local Research Ethics Committees (further details can be found at https://www.bristol.ac.uk/alspac/). Our study’s sample (N = 4583) was children with valid data on inflammatory markers at age 9 (the only time in childhood they were measured in ALSPAC) who did not report an infection at the time of blood collection or during the preceding week.

### Measures

#### Peer victimisation (age 8)

The child-reported Bullying and Friendship Interview Schedule [23], a measure of high inter-rater reliability and predictive ability, was used to assess peer victimisation at age 8. During the interview, children rated their experience of victimisation over the past six months based on five questions for overt victimisation and four questions for relational victimisation. If a child reported an experience of peer victimisation, they were asked how frequently it had occurred: 1 to 3 times (infrequently), ≥ 4 times (frequently), or at least once a week (very frequently). In our study, overt or relational victimisation was deemed present if experienced frequently or very frequently [51].

#### Inflammation (age 9)

In ALSPAC, inflammation in childhood was measured at age 9 years with blood levels of interleukin 6 (IL-6) and C-reactive protein (CRP). IL-6 is a pro-inflammatory cytokine and CRP, whose production is stimulated by IL-6, is an inflammatory protein, and both are commonly used as inflammatory markers in behavioural research [15]. During clinic visits, blood samples were obtained from non-fasting ALSPAC participants and were immediately spun and frozen at − 80 °C, with no evidence of freeze–thaw cycles during storage. Enzyme-linked immunosorbent assay was used to measure IL-6 (pg/mL) (R&D Systems, Abingdon, UK) and automated particle-enhanced immunoturbidimetric assay was used to measure high-sensitivity CRP (mg/L) (Roche, UK). All interassay coefficients of variation were less than 5%. IL-6 and CRP measures were log-transformed in the analysis to correct for skewness.

#### Working memory (age 10)

Working memory was measured using the computer-based Counting Span Task [10] which requires the simultaneous processing and storage of information. During the processing phase, the child was shown a series of screens on a computer, each displaying red and blue dots. On each screen, the child had to count the number of red dots and say the count out loud. The child was presented with (i) two practice sets of two screens, (ii) three sets of two screens, (iii) three sets of three screens, (iv) three sets of four screens, and (v) three sets of five screens. A set consists of a sequence of screens. For example, a set of two screens means the child will see two different screens, one after the other, and count the red dots on each. During the storage component, the child had to recall the number of red dots seen on each screen after each set in the order they were presented within that set. Each child worked through every set regardless of their overall performance. Working memory was calculated as the number of correctly recalled sets, weighted by the number of screens within each set, with a maximum score of 5 (i.e., all correct). The test produces wo scores: (i) a global score comprising the number of correct trials and (ii) a span score, the main outcome measure for this task, which is what was used in this study. The span score was selected as the primary outcome measure as it captures the child’s ability to handle increasing cognitive load, providing a more sensitive indicator of working memory capacity compared to the global score.

#### Reading ability (age 9)

Reading ability was assessed using the Neale Analysis of Reading Ability (NARA II) [37]. The child was asked to read short passages of text and answer comprehension questions about the story they had just read. The test includes three stories of increasing difficulty (levels 1–3). A practice trial was administered first, during which the child read a practice passage. If the child made more than 17 reading errors on the practice passage, the tester did not administer the comprehension questions and moved directly to the level 1 story. If fewer than 17 errors were made, the tester proceeded with the comprehension questions for the practice passage. Most children moved on to the level 2 story unless they appeared to have struggled with reading the practice passage. If the child made fewer than three reading errors on the level 2 story, the tester moved to level 3. However, if the child made three or more errors on the level 2 story, the tester administered the comprehension questions for that level but then moved down to the level 1 story. Comprehension questions were asked immediately after the child finished reading, and the child had 10 to 12 s to respond to each question. The test produces three measures: reading speed (words read per minute), reading accuracy (number of reading errors), and comprehension (correct responses to comprehension questions), and we used all three in the current study.

#### Spelling (age 9)

Spelling was assessed with the age 9 spelling test developed by Nunes and Bryant [39]. For this test, the child was asked to spell 15 words that increased in difficulty. The tester read each word out loud and used it in a sentence, and the child wrote down the word. The test’s score, used in this study, is the number of correctly spelt words.

#### Response inhibition (age 10)

Response inhibition was measured using the stop signal task [27], assessing the inhibition of a requested body movement. Each child was seated in front of a computer monitor, with their two index fingers placed on two buttons: one labelled X and one labelled O. Two types of trials were conducted: primary task trials and stop signal trials. During the primary task trials, the child was asked to focus on a smiley face shown in the centre of the screen. An X or O would then be shown on the screen and the child had to press the corresponding button as quickly as possible. Thirty trials in total were completed and a mean reaction time was calculated. This was used to calculate a tone delay in subsequent trials. The stop signal trials were identical to the primary task trials, but a bleep (stop signal) was heard randomly after the X or O appeared (go signal). If the stop signal was not heard, the child was asked to press the corresponding X or O button. However, if the stop signal sounded, the child was told to refrain from pressing the button, inhibiting their response. The signal sounded on random trials at either 150 ms or 250 ms before the child’s reaction time (calculated in primary task trials). This second block of trials involved 24 practice trials, with 8 primary task trials and 16 stop signal trials (5 at each of the two intervals). Two more blocks of trials were completed (experimental blocks) which consisted of 48 trials in each,16 trials were with stop signals. The measures produced from this task include the number of primary trials correct in the experimental block, the mean reaction time in primary trials in the experimental block, the number of stop signal trials correct at 250 ms delay, and the number of stop signal trials correct at 150 ms delay. The latter two measures were used here given the study aims.

#### Selective attention (age 11)

Selective attention was measured with the Sky Search measure from the Test of Everyday Attention for Children (TEA-Ch) [41]. The child’s ability to efficiently filter important information was assessed by having them circle as many pairs of identical spaceships among other non-identical spaceships as quickly as possible without making any errors. Before starting the task, the child worked through a practice sheet. The tester then went through any errors with the child. After the practice, the child completed the same task using a sheet containing more pairs of spaceships (where 20 pairs were identical). This task was also repeated with a sheet containing only the 20 identical pairs of spaceships but arranged in a different way from the original sheet. The purpose of this was to measure how quickly the child completed the task, so that motor performance could be adjusted for. The time taken to circle the identical spaceships, recorded in seconds, is the task’s primary outcome, and is what we used in this study.

#### Attentional control (age 11)

Attentional control was measured with the Opposite Worlds measure from the Test of Everyday Attention for Children (TEA-Ch) [41]. Here, the child was shown a list of the numbers 1 and 2 (24 numbers in total) under two different conditions. The first condition was the ‘same world’ (control) condition in which the child had to follow the tester’s finger and read the numbers that were pointed to out loud as quickly as possible. The tester kept their finger by the number until the child had given the correct response. In the ‘opposite world’ condition, the task was administered in the same way, but the child had to call out ‘two’ when the tester pointed to a 1 and ‘one’ when the tester pointed to a 2. The child was given a demonstration of each condition and had a practice attempt at each. There were four test trials in total (two same-world trials and two opposite-world trials). The task is scored by the mean time taken (in seconds) to correctly read the numbers out loud. The opposite world task score was used here given the study aims.

#### Confounders

Several potential confounders, associated with peer victimisation, inflammation and cognitive outcomes, were controlled for. These included ethnicity (at birth) [43, 52], sex (at birth) [9, 31], body mass index (BMI) (at age 9) [5, 26], socioeconomic status (parental education and social class) [16, 36, 48], maternal depression [38], and stressful life events experienced—both family and child-specific [28, 32]. Given the very low number of ethnic minority families in ALSPAC, ethnicity was dichotomously coded as white and non-white. Sex was coded as female and male. Maternal education (at 32 weeks of pregnancy) was highest qualification obtained, including CSE, vocational, O level, A level, and degree, with higher values representing higher levels of education. Paternal social class (at 32 weeks of pregnancy) was comprised of the categories: I, II, III (non-manual), III (manual), IV, and V, with higher values representing lower social class. The Edinburgh Postnatal Depression Scale (EPDS) was used to measure maternal depression at 32 weeks of pregnancy, with a higher score indicating a greater level of depression [29]. Stress experienced by the mother and family across the child’s life and as near as possible to age 8 years (when peer victimisation was measured) was assessed using a checklist of 43 stressful life events, completed by the mother when the child was 21 months (covering events since the child was age 8 months), 33 months (events since age 18 months), 47 months (events since age 33 months), 61 months (events since age 47 months), and 73 months (events since age 61 months) [6]. The number of events at each time-point was summed to produce a total number of stressful life events that occurred up until the child was about 6 years of age. Child-specific stressful life events were measured when the child was about age 8 using a checklist completed by the mother, containing a list of 17 potentially upsetting events for the child that may have occurred since their 7th birthday. Each event was dichotomously scored as having happened or not, and events were summed to produce the total number of stressful life events experienced by the child from age 7 to age 8.

### Analytic strategy

Analyses were conducted in SPSS and AMOS version 29. Descriptive statistics summarised the characteristics of the sample. Bivariate correlations were calculated between the main variables using Pearson’s correlation coefficients. Missingness was handled with regression imputation using maximum likelihood estimates. Mediation models were fitted before and after adjusting for potential confounders. Bootstrapping was used, with 5000 usable bootstrap samples obtained for each model.

## Results

### Descriptive statistics

The descriptive statistics of the sample can be seen in Table 1. The sample mostly contained children of white ethnicity (96%) but was relatively equally divided by sex (49% female). More children were involved in overt (34.2%) compared to relational bullying (16.8%), as expected. Among the children involved in bullying, the majority identified themselves as pure victims of either overt bullying (27.4%) or relational bullying (14.3%). A smaller group identified as bully-victims, meaning they both bullied others and were victims of bullying themselves. The most frequently reported level of maternal education was O level (35.1%), while the most frequently reported paternal social class was II (36.4%).

**Table 1:** Descriptive statistics of the sample (N = 4583)

	N	%
*Categorical variables*		
Sex (Female)	2243	49.0
Ethnicity (White)	3983	96.0
*Maternal education*		
CSE	552	13.1
Vocational	330	7.8
O level	1476	35.1
A level	1135	27.0
Degree	711	16.9
*Paternal social class*		
I	490	12.6
II	1412	36.4
III (non-manual)	478	12.3
III (manual)	1102	28.4
IV	309	8.0
V	84	2.2
*Overt bullying status*		
Pure bully	42	1.1
Pure victim	1047	27.4
Bully-victim	220	5.7
Neutral	2518	65.8
*Relational bullying status*		
Pure bully	26	0.7
Pure victim	538	14.3
Bully-victim	66	1.8
Neutral	3123	83.2

	N	Mean (SD), Min–Max
*Continuous variables*		
Maternal stressful life events	2534	11.59 (4.35)
Child-specific stressful life events	2856	2.03 (1.21)
Interleukin 6 (pg/mL)	4583	1.21 (1.47)
C-reactive protein (mg/L)	4583	0.62 (1.93)
BMI	4531	17.57 (2.76)
Maternal depression	4137	6.70 (4.86)
Working memory	3866	3.43 (0.85), 0–5
Reading speed	4087	105.29 (12.56), 69–131
Rading accuracy	4098	103.90 (13.60), 69–131
Reading comprehension	4098	100.22 (11.88), 69–131
Spelling	4540	10.24 (3.45), 0–15
Response inhibition (250 ms delay)	3858	13.66 (2.68), 0–16
Response inhibition (150 ms delay)	3858	12.10 (3.07), 0–16
Selective attention	3883	3.56 (1.26), .15–32.00
Attentional control	3758	18.45 (1.35), 1–19

### Bivariate correlations

Table 2 shows the bivariate correlations between the main variables. As can be seen, peer victimisation correlated with both IL-6 and CRP, and was inversely correlated with working memory, all outcomes of reading ability (speed, accuracy, and comprehension), and spelling. It was also inversely correlated with response inhibition (number of stop signal trials correct at both 250 ms and 150 ms delay) and selective attention. Peer victimisation did not significantly correlate with attentional control, however. Both IL-6 and CRP were negatively correlated with working memory. Both IL-6 and CRP were negatively correlated with reading accuracy and reading comprehension but only CRP was negatively correlated with reading speed. Neither IL-6 nor CRP significantly correlated with spelling, response inhibition, attentional control, or selective attention.

**Table 2:** Bivariate correlations of main variables

	PV	IL-6	CRP	WM	S	RS	RA	RC	RI 250	RI 150	SA	AC
PV	1											
IL-6	.040*	1										
CRP	.034*	.452**	1									
WM	− .044*	− .048**	− .049**	1								
S	− .088**	− .012	− .017	.296**	1							
RS	− .058**	− .021	− .048**	.292**	.633**	1						
RA	− .066**	− .053**	− .050**	.324**	.791**	.749**	1					
RC	− .058**	− .065**	− .058**	.320**	.659**	.658**	.813**	1				
RI 250	− .057**	− .012	− .016	.117**	.104**	.105**	.122**	.138**	1			
RI 150	− .051**	− .022	− .013	.080**	.057**	.068**	.077**	.084**	.718**	1		
SA	.039*	.011	− .002	− .148**	− .207**	− .157**	− .175**	− .161**	− .123**	− .088**	1	
AC	− .034	− .003	− .015	.185**	.252**	.230**	.264**	.255**	.138**	.119**	− .345**	1

Peer Victimisation (PV); Working Memory (WM); Spelling (S); Reading Speed (RS); Reading Accuracy (RA); Reading Comprehension (RC); Response Inhibition, 250 ms (RI 250); Response Inhibition, 150 ms (RI 150); Selective Attention (SA); Attentional Control (AC) (*p < .05; **p < .01)

### Regression models

Regression coefficients before and after adjusting for confounders can be seen in Table 3 for models where indirect effects remained significant after adjustment (results for all other models in the Supplementary Material). Mediation effect size (ES) was calculated using the Proportion Mediated (PM) measure [30], i.e., the proportion of the total effect attributable to the indirect effect. The PM is a number between 0 and 1, whereby a greater value represents a larger mediating effect.

**Table 3:** Unstandardised regression coefficients (alongside standard errors and 95% CI) for mediation models where indirect effects remained significant after adjustment

Unadjusted model				Adjusted model		
*Peer Victimisation (PV) → CRP → Working Memory (WM), age 10*						
PV → WM	− .092***	.028	− .149, − .034	− .075**	.028	− .132, − .018
PV → CRP	.046*	.018	.008, .083	.030	.016	− .003, .063
CRP → WM	− .081***	.023	− .127, − .037	− .081**	.026	− .133, − .033
Indirect effect	− .004**	.002	− .009, − .001	− .002*	.002	− .007, .000
Total effect	− .096***	.029	− .153, − .037	− .078**	.029	− .134, − .020
*Peer Victimisation (PV) → IL-6 → Working Memory (WM), age 10*						
PV → WM	− .091***	.028	− .148, − .033	− .075**	.028	− .133, − .017
PV → IL-6	.039**	.013	.013, .065	.029*	.013	.003, .053
IL-6 → WM	− .107***	.031	− .171, − .047	− .087**	.032	− .152, − .026
Indirect effect	− .004**	.002	− .009, − .001	− .002*	.002	− .006, .000
Total effect	− .095***	.029	− .152, − .037	− .078**	.029	− .135, − .020
*Peer Victimisation (PV) → CRP → Reading Speed (RS), age 9*						
PV → RS	− 1.899***	.429	− 2.760, − 1.061	− 1.473***	.405	− 2.289, − .664
PV → CRP	.046*	.018	.008, .083	.030	.016	− .003, .063
CRP → RS	− 1.161***	.352	− 1.870, − .494	− .978**	.375	− 1.708, − .267
Indirect effect	− .053**	.027	− .120, − .011	− .029*	.020	− .084, .000
Total effect	− 1.952***	.432	− 2.816, − 1.113	− 1.502***	.410	− 2.318, − .698
*Peer Victimisation (PV) → CRP → Reading Accuracy (RA), age 9*						
PV → RA	− 2.329***	.465	− 3.183, − 1.428	− 1.868***	.435	− 2.695, − 1.039
PV → CRP	.045*	.018	.008, .083	.030	.016	− .003, .063
CRP → RA	− 1.286***	.382	− 2.022, − .529	− .928*	.403	− 1.736, − .145
Indirect effect	− .059*	.030	− .133, − .012	− .028*	.021	− .086, .000
Total effect	− 2.388***	.449	− 3.243, − 1.492	− 1.896***	.423	− 2.726, − 1.071
*Peer Victimisation (PV) → IL-6 → Reading Accuracy (RA), age 9*						
PV → RA	− 2.319***	.465	− 3.173, − 1.415	− 1.874***	.485	− 2.699, − 1.043
PV → IL-6	.039**	.013	.013, .065	.028*	.013	.003, .053
IL-6 → RA	− 1.840***	.515	− 2.817, − .845	− .990	.500	− 1.946, .001
Indirect effect	− .072***	.032	− .152, − .023	− .028*	.020	− .082, − .001
Total effect	− 2.391***	.449	− 3.243, − 1.502	− 1.902***	.423	− 2.737, − 1.080
*Peer Victimisation (PV) → IL-6 → Reading Comprehension (RC), age 9*						
PV → RC	− 1.782***	.406	− 2.540, − .998	− 1.386***	.368	− 2.103, − .665
PV → IL-6	.039**	.013	.013, .065	.029*	.013	.003, .053
IL-6 → RC	− 2.003***	.450	− 2.887, − 1.138	− 1.009*	.424	− 1.831, − .193
Indirect effect	− .079**	.033	− .155, − .025	− .029*	.019	− .077, − .003
Total effect	− 1.861***	.395	− 2.623, − 1.080	− 1.414***	.363	− 2.135, − .694
*Peer Victimisation (PV) → CRP → Selective Attention (SA), age 11*						
PV → SA	.137***	.042	.057, .225	.097*	.041	.018, .180
PV → CRP	.046*	.018	.008, .083	.030	.016	− .003, .063
CRP → SA	− .008	.034	− .073, .058	.083*	.038	.014, .154
Indirect effect	.000	.002	− .004, .003	.002*	.002	.000, .008
Total effect	.137***	.043	.057, .225	.100*	.040	.022, .181
*Peer Victimisation (PV) → IL-6 → Selective Attention (SA), age 11*						
PV → SA	.137***	.042	.057, .224	.097*	.041	.019, .179
PV → IL-6	.040**	.013	.013, .066	.029*	.013	.003, .054
IL-6 → SA	.032	.046	− .055, .127	.121**	.047	.040, .205
Indirect effect	.001	.002	− .002, .007	.003*	.002	.000, .009
Total effect	.139***	.043	.059, .227	.101*	.040	.023, .183

*p< .05; **p< .01; ***p < .001

There was a significant indirect effect of peer victimisation on working memory via both IL-6 (adjusted model: b = − 0.002, 95% CI [− 0.006, 0.000], *p* < 0.05, ES = 0.026) and CRP (adjusted model: b = − 0.002, 95% CI [− 0.007, 0.000], *p* < 0.05, ES = 0.026). A significant indirect effect of peer victimisation on reading accuracy was also found via both IL-6 (adjusted model: b = − 0.028, 95% CI [− 0.082, − 0.001], *p* < 0.05, ES = 0.015) and CRP (adjusted model: b = − 0.028, 95% CI [− 0.086, 0.000], *p* < 0.05, ES = 0.015). These results demonstrate that peer victimisation is associated with poorer working memory and worse reading ability partly via inflammation.

Inflammation also partly mediated the effect of peer victimisation on selective attention. This was observed for IL-6 (adjusted model: b = 0.003, 95% CI [0.000, 0.009], *p* < 0.05, ES = 0.030) and CRP (adjusted model: b = 0.002, 95% CI [0.000, 0.008], *p* < 0.05, ES = 0.020). There was also some mediator specificity, but always with reading. Only CRP had a mediating role in the effect of peer victimisation on reading speed (adjusted model: b = − 0.029, 95% CI [− 0.084, 0.000], *p* < 0.05, ES = 0.019). Only IL-6 mediated the effect of peer victimisation on reading comprehension (adjusted model: b = − 0.029, 95% CI [− 0.077, − 0.003], *p* < 0.05, ES = 0.021).

## Discussion

The current study suggests that peer victimisation may produce poor cognitive outcomes in childhood via inflammation (as measured by IL-6 and CRP). Nonetheless, effects were small and there seems to be a great degree of specificity involved. For example, inflammation did not mediate the effects of peer victimisation on spelling, attentional control or response inhibition. However, both inflammatory markers partially mediated the effects of peer victimisation on working memory, reading accuracy, and selective attention. The finding that CRP was associated with poorer working memory contrasts to the null findings of Cullen et al. [14]. However, the current study used blood-derived CRP, rather than salivary CRP, which could be responsible for this difference [17]. The difference could also be due to power (their sample was just over 100 adolescents) or sample characteristics: ours is a general population sample of primary school age children, whereas theirs was a sample of adolescents at risk of psychopathology.

Importantly, our study demonstrates robust effects of peer victimisation on several aspects of a child’s cognitive function, even after controlling for multiple potential confounders, including other stressors experienced since birth. This highlights the specificity and saliency of the effects of peer victimisation in childhood. Peer victimisation has been associated with poorer cognition in childhood and abnormalities in several brain networks [49], but the findings of the current study indicate that experiencing peer victimisation could biologically impair cognitive development at this age. Further research on the role of peer victimisation in child cognitive functioning could subsequently investigate potential chain mediation models involving both inflammatory and neural changes. Uniquely for reading outcomes there was also some mediator specificity. Only CRP mediated the effect of peer victimisation on reading speed, whereas only IL-6 mediated its effect on reading comprehension. This emphasises the importance for future related research to use multiple inflammatory markers and multiple cognitive outcomes.

Although our results are preliminary, we ought to discuss those pertaining to reading, the cognitive outcome most consistently associated with the mediation model tested in this study. Reverse causation is plausible here given the evidence that those who have reading difficulties tend to be bullied more. Another possibility may be that what we see here is the result of the classic withdrawal and sickness behaviour that inflammation typically produces. That is, victims of bullying tend to be anxious and prone to developing avoidance behaviours, such as not reading aloud in class, which in turn limits their practice in speed and accuracy. In fact, the NARA test, used in this study, includes reading aloud. Therefore, according to this hypothesis, victimised children either were too anxious or had practiced too little.

In general, interpretations of our findings are limited because observational data preclude causal claims. While the ages at which the main variables were measured allow for a causal pathway to be tested, the possibility of reverse causation cannot be overlooked. For example, it is unknown whether cognitive difficulties were present before measures of peer victimisation or inflammation were taken. A recent study, for example, showed that impaired working memory predicted experiences of peer victimisation among adolescents with ADHD [54]. A truly longitudinal design across the childhood years with repeated measures of peer victimisation, inflammatory markers and cognitive function would alleviate some of these concerns, and we urge researchers to consider this suggestion in future studies. There are also concerns about the validity of some of our measures. For example, stressful events experienced by the child were reported by the mother. Furthermore, peer victimisation, generally characterised as a multidimensional rather than a singular construct, was measured rather crudely.

Despite these limitations, our findings have important implications. During childhood and adolescence, the developing brain is particularly susceptible to the effects of stressors, especially in slow-developing frontal regions involved in cognition [3, 21]. Experiencing a stressor such as peer victimisation at this age can be highly detrimental to cognitive development, especially as peer victimisation tends to be chronically occurring [44]. While previous research demonstrated concurrent associations between peer victimisation and inflammation with cognitive outcomes in youth [14, 34], our prospective findings offer a stronger basis for suggesting that peer victimisation impairs brain development during childhood via inflammation, likely resulting in lasting effects on cognition.

## Conclusion

This is the first study using ALSPAC that explores the role of inflammation in mediating the effect of peer victimisation on cognition in children. It shows clear evidence that inflammation can explain part of the detrimental effect of peer victimisation on several, but specific, cognitive functions in childhood. Although this indirect effect is small it suggests a neurobiological mechanism linking peer victimisation and child cognitive functioning.

## Supplementary Information

Below is the link to the electronic supplementary material.

Supplementary file1 (DOCX 22 KB)
