# Supplementary material for: The effect of peer victimisation on cognitive development in childhood: evidence for mediation via inflammation
Source: Soc Psychiatry Psychiatr Epidemiol. 2025 Mar 17;60(11):2553–62. doi: 10.1007/s00127-025-02836-0 (PMC12572086; doi:10.1007/s00127-025-02836-0)
Supplement: Supplementary file 1 — Supplementary file1 (DOCX 22 KB) [file 127_2025_2836_MOESM1_ESM.docx]

**Supplementary material**

| **Peer Victimisation (PV) 🡪 IL-6 🡪 Spelling Score**  ***Unadjusted model*** | | | |
| --- | --- | --- | --- |
| PV 🡪 Spelling | -.835*** | .124 | -1.074, -.592 |
| PV 🡪 IL-6 | .040** | .013 | .013, .066 |
| IL-6 🡪 Spelling | -.073 | .138 | -.343, .197 |
| Indirect effect | -.003 | .006 | -.016, .008 |
| Total effect | -.838*** | .125 | -1.079, .000 |
| ***Adjusted model*** | | | |
| PV 🡪 Spelling | -.702*** | .119 | -.934, -.464 |
| PV 🡪 IL-6 | .029* | .013 | .004, .054 |
| IL-6 🡪 Spelling | -.074 | .137 | -.338, .195 |
| Indirect effect | -.002 | .004 | -.014, .005 |
| Total effect | -.705*** | .121 | -.937, -.465 |
| **Peer Victimisation (PV) 🡪 CRP 🡪 Spelling Score**  ***Unadjusted model*** | | | |
| PV 🡪 Spelling | -.831*** | .124 | -1.070, -.590 |
| PV 🡪 CRP | .046* | .018 | .008, .083 |
| CRP 🡪 Spelling | -.092 | .102 | -.285, .116 |
| Indirect effect | -.004 | .005 | -.018, .004 |
| Total effect | -.835*** | .125 | -1.077, -.593 |
| ***Adjusted model*** | | | |
| PV 🡪 Spelling | -.696*** | .119 | -.932, -.458 |
| PV 🡪 CRP | .030 | .016 | -.003, .063 |
| CRP 🡪 Spelling | -.213 | .110 | -.435, .005 |
| Indirect effect | -.006 | .005 | -.022, .000 |
| Total effect | -.703*** | .121 | -.934, -.462 |
| **Peer Victimisation (PV) 🡪 IL-6 🡪 Words per Minute (WpM)**  ***Unadjusted model*** | | | |
| PV 🡪 WpM | -1.921*** | .429 | -2.787, -1.074 |
| PV 🡪 IL-6 | .039** | .013 | .013, .065 |
| IL-6 🡪 WpM | -.629 | .476 | -1.536, .285 |
| Indirect effect | -.025 | .021 | -.081, -.005 |
| Total effect | -1.946 | .431 | -2.815, -1.104 |
| ***Adjusted model*** | | | |
| PV 🡪 WpM | -1.509*** | .406 | -2.326, -.698 |
| PV 🡪 IL-6 | .028* | .013 | .003, .053 |
| IL-6 🡪 WpM | .079 | .466 | -.786, .978 |
| Indirect effect | .002 | .014 | -.023, .036 |
| Total effect | -1.506*** | .410 | -2.327, -.700 |
| **Peer Victimisation (PV) 🡪 CRP 🡪 Comprehension**  ***Unadjusted model*** | | | |
| PV 🡪 Comprehension | -1.788*** | .406 | -2.555, -1.011 |
| PV 🡪 CRP | .045* | .018 | .007, .082 |
| CRP 🡪 Comprehension | -1.333*** | .333 | -1.997, -.650 |
| Indirect effect | -.060* | .030 | -.131, =.012 |
| Total effect | -1.848*** | .395 | -2.611, -1.064 |
| ***Adjusted model*** | | | |
| PV 🡪 Comprehension | -1.374*** | .368 | -2.091, -.652 |
| PV 🡪 CRP | .029 | .016 | -.003, .063 |
| CRP 🡪 Comprehension | -.902** | .341 | -1.594, -.203 |
| Indirect effect | -.027 | .019 | -.077, .000 |
| Total effect | -1.400*** | .363 | -2.119, -.677 |
| **Peer Victimisation (PV) 🡪 CRP 🡪 Response Inhibition (Stop Signal 250ms delay) (SS250)**  ***Unadjusted model*** | | | |
| PV 🡪 SS250 | -.386*** | .089 | -.579, -.201 |
| PV 🡪 CRP | .045* | .018 | .007, .082 |
| CRP 🡪 SS250 | -.072 | .073 | -.214, .061 |
| Indirect effect | -.003 | .004 | -.014, .002 |
| Total effect | -.390*** | .096 | -.584, -.203 |
| ***Adjusted model*** | | | |
| PV 🡪 SS250 | -.354*** | .089 | -.548, -.167 |
| PV 🡪 CRP | .029 | .016 | -.003, .063 |
| CRP 🡪 SS250 | -.101 | .082 | -.262, .056 |
| Indirect effect | -.003 | .003 | -.013, .001 |
| Total effect | -.357*** | .096 | -.552, -.174 |
| **Peer Victimisation (PV) 🡪 IL-6 🡪 Response Inhibition (Stop Signal 250ms delay) (SS250)**  ***Unadjusted model*** | | | |
| PV 🡪 SS250 | -.389*** | .089 | -.580, -.203 |
| PV 🡪 IL-6 | .039** | .013 | .013, .065 |
| IL=6 🡪 SS250 | -.065 | .099 | -.270, .128 |
| Indirect effect | -.003 | .004 | -.013, .004 |
| Total effect | -.392*** | .096 | -.586, -.206 |
| ***Adjusted model*** | | | |
| PV 🡪 SS250 | -.357*** | .089 | -.550, -.173 |
| PV 🡪 IL-6 | .029* | .013 | .003, .053 |
| IL-6 🡪 SS250 | -.053 | .102 | -.266, .146 |
| Indirect effect | -.002 | .003 | -.010, .004 |
| Total effect | -.359*** | .096 | -.553, -.176 |
| **Peer Victimisation (PV) 🡪 CRP 🡪 Response Inhibition (Stop Signal 150ms delay) (SS150)**  ***Unadjusted model*** | | | |
| PV 🡪 SS150 | -.395*** | .102 | -.620, -.184 |
| PV 🡪 CRP | .045* | .018 | .008, .082 |
| CRP 🡪 SS150 | -.063 | .084 | -.225, .097 |
| Indirect effect | -.003 | .001 | -.014, .003 |
| Total effect | -.398*** | .109 | -.622, -.186 |
| ***Adjusted model*** | | | |
| PV 🡪 SS150 | -.367*** | .102 | -.589, -.154 |
| PV 🡪 CRP | .029 | .016 | -.003, .063 |
| CRP 🡪 SS150 | -.124 | .095 | -.304, .065 |
| Indirect effect | -.004 | .004 | -.015, .001 |
| Total effect | -.371*** | .109 | -.593, -.158 |
| **Peer Victimisation (PV) 🡪 IL-6 🡪 Response Inhibition (Stop Signal 150ms delay) (SS150)**  ***Unadjusted model*** | | | |
| PV 🡪 SS150 | -.392*** | .102 | -.618, -.182 |
| PV 🡪 IL-6 | .039** | .013 | .012, .065 |
| IL-6 🡪 SS150 | -.159 | .113 | -.396, .078 |
| Indirect effect | -.006 | .005 | -.020, .002 |
| Total effect | -.398*** | .109 | -.620, -.184 |
| ***Adjusted model*** | | | |
| PV 🡪 SS150 | -.366*** | .102 | -.588, -.152 |
| PV 🡪 IL-6 | .028* | .013 | .003, .053 |
| IL-6 🡪 SS150 | -.189 | .117 | -.432, .053 |
| Indirect effect | -.005 | .005 | -.018, .001 |
| Total effect | -.371*** | .109 | -.595, -.159 |
| **Peer Victimisation (PV) 🡪 CRP 🡪 Attentional Control (Opposite World OW)**  ***Unadjusted model*** | | | |
| PV 🡪 OW | -.177** | .044 | -.205, -.033 |
| PV 🡪 CRP | .046* | .018 | .008, .083 |
| CRP 🡪 OW | -.037 | .036 | -.109, .033 |
| Indirect effect | -.002 | .002 | -.007, .001 |
| Total effect | -.119** | .044 | -.206, -.034 |
| ***Adjusted model*** | | | |
| PV 🡪 OW | -.067 | .044 | -.156, .017 |
| PV 🡪 CRP | .030 | .016 | -.003, .063 |
| CRP 🡪 OW | -.059 | .040 | -.137, .016 |
| Indirect effect | -.002 | .002 | -.007, .000 |
| Total effect | -.069 | .044 | -.158, .016 |
| **Peer Victimisation (PV) 🡪 IL-6 🡪 Attentional Control (Opposite World OW)**  ***Unadjusted model*** | | | |
| PV 🡪 OW | -.118** | .044 | -.205, -.033 |
| PV 🡪 IL-6 | .039** | .013 | .013, .065 |
| IL-6 🡪 OW | -.004 | .049 | -.091, .083 |
| Indirect effect | .000 | .002 | -.004, .004 |
| Total effect | -.118** | .044 | -.204, -.033 |
| ***Adjusted model*** | | | |
| PV 🡪 OW | -.068 | .044 | -.157, .016 |
| PV 🡪 IL-6 | .029* | .013 | .003, .053 |
| IL-6 🡪 OW | -.001 | .050 | -.091, .089 |
| Indirect effect | .000 | .001 | -.003, .003 |
| Total effect | -.068 | .044 | -.158, .016 |
